# Comprehensive Assessment of Cardiac Function in Breast Cancer Patients: Integrating Multiparametric Speckle Tracking Imaging and Conventional Echocardiography Parameters

**DOI:** 10.7759/cureus.57791

**Published:** 2024-04-07

**Authors:** Marina Leitman, Shmuel Fuchs, Oran Tzuman, Rotem Merose, Sara Shimoni

**Affiliations:** 1 Cardiology, Shamir Medical Center, Zerifin, ISR; 2 Sackler School of Medicine, Tel Aviv University, Tel Aviv, ISR; 3 Cardiology, Kaplan Medical Center, Rehovot, ISR; 4 Medicine, Hadassah Medical School, Hebrew University, Jerusalem, ISR

**Keywords:** chemotherapy, cardioprotection, breast cancer patients, automatic ejection fraction, cardiotoxicity, myocardial work index, global longitudinal strain

## Abstract

Purpose

The purpose of this study is to comprehensively evaluate the role of different echocardiography parameters in breast cancer patients undergoing chemotherapy. While echocardiography examination with calculation of ejection fraction (EF), is pivotal for patient monitoring, its operator dependence and insensitivity to subtle changes in left ventricular (LV) contractility present challenges. Global longitudinal strain (GLS), derived from speckle tracking, is more sensitive and stable than EF. Our research aimed to delineate supplementary echocardiography measurements beneficial for the cardiological monitoring of breast cancer patients.

Methods

Patients were followed up with echocardiography at baseline, during, and after the chemotherapy. Conventional echocardiography and multiple speckle tracking imaging parameters including myocardial work index, atrial strain, twist, and automatic EF were investigated.

Results

A total of 25 patients were recruited. A subset (15/25) exhibited pronounced GLS reduction, associated with decreased EF and altered cardiac mechanics. Patients with unchanged GLS were often hypertensive and on specific medications, in particular angiotensin-converting enzyme inhibitors (ACE inhibitors)/angiotensin II receptor blockers (ARBs), potentially indicating protective effects. Despite stability in other parameters, GLS and EF sensitivity highlight their importance. A strong correlation between manual and automated EF measurement methods was also observed.

Conclusion

Despite the small sample size, across diverse echocardiography parameters, GLS and EF are primarily affected by chemotherapy. Hypertensive individuals exhibited lower susceptibility to chemotherapy-induced damage, likely attributed to the cardioprotective properties of ACE inhibitors and angiotensin II receptor blockers. A strong correlation between automatic and Simpson-based EF was found.

## Introduction

Chemotherapeutic agents utilized in the treatment of oncological patients are known to induce cardiac toxicity, which leads to cardiac dysfunction depending on the specific agent used and the individual cardiac reserve of the patients. The cardiac effects of chemotherapeutic agents have been extensively studied in breast cancer patients. Echocardiography is a primary modality employed for a thorough follow-up of these patients, with calculated ejection fraction (EF) serving as a key indicator of cardiac function [1]. However, the conventional method of assessing EF is operator-dependent, potentially lacking accuracy, and can overlook subtle changes in left ventricular (LV) contractility [2]. Temporal variability of echocardiography-derived EF has been reported to exceed 10% [3]. To address these limitations, advanced echocardiographic techniques such as speckle tracking imaging have emerged, enabling precise quantitative evaluation of cardiac function. Global longitudinal strain (GLS), derived from speckle tracking imaging, has gained prominence in cardiac oncology because of its heightened sensitivity and stability compared to EF. This is particularly crucial in patients with preserved LV function (EF of 50-55%). Studies have shown that a more 2% reduction in GLS predicts cardiotoxicity with a sensitivity of 79% and specificity of 82% [4]. Subsequently, numerous investigations have explored diverse speckle-tracking parameters for assessing cardiac toxicity. Notably, radial and circumferential strains were found to decrease during chemotherapy [5,6]. Chemotherapy-induced alterations in left ventricular torsion and twisting velocity have also been documented [7]. Additionally, the impact of chemotherapy on right ventricular function has been observed, either as a consequence of preexisting dysfunction or cardiotoxicity [8]. Although the prognostic value of the right ventricular function in oncological patients remains uncertain, the evaluation of right ventricular function, as well as the right atrial area, has become a routine component of assessing right heart function in these patients [9]. Diastolic dysfunction has been extensively investigated in the context of chemotherapy [10-13]. While a reduction in e' velocity has been noted in cancer patients undergoing chemotherapy [13-15], diastolic parameters, in general, have not been proven to be predictive of chemotherapy-associated systolic dysfunction. Parameters such as the E/A ratio and E/e' ratio, which are load-dependent, can be affected by chemotherapy-induced nausea and vomiting. Prolongation of the myocardial performance index post-chemotherapy has been reported, but it lacks consistent reproducibility [16-18]. Given the challenges in evaluating diastolic function during chemotherapy, efforts have been directed toward identifying more accurate methods for assessing left ventricular filling pressure. Notably, left atrial size and function have been recognized as potential indicators of left ventricular filling pressure, with left atrial strain emerging as a promising parameter for assessing left atrial function [19]. In this study, we aim to comprehensively assess the role of various echocardiography parameters in evaluating breast cancer patients undergoing chemotherapy. Through this thorough exploration, we seek to enhance our understanding of the multifaceted cardiac effects of chemotherapy and its implications for patient care.

## Materials and methods

This prospective study was approved by the Ethics Local Helsinki Committee at the Shamir and Kaplan Medical Centers for the conduct of clinical studies. All methods were conducted while following relevant guidelines and regulations. The participants were recruited from both Shamir Medical Center and Kaplan Medical Center. Before inclusion in the study, all patients provided their informed consent to participate. All patients underwent echocardiography examination according to the judgment of the attending physician, before, during, and after chemotherapy. Both conventional and advanced speckle-tracking imaging parameters were systematically collected and subjected to thorough analysis. Demographic and clinical data were meticulously acquired in alignment with the information documented in hospital records. Patients who had previously undergone chemotherapy and individuals unable to provide informed consent were excluded from the study.

Echocardiography examination

The standard echocardiography assessment adhered to prevailing recommendations. The VIVID E95 GE echocardiography machine (GE Healthcare, Chicago, IL) was employed for the study, and all examinations were digitally recorded. The evaluation encompassed linear and volumetric measurements, as well as an analysis of diastolic function, including parameters such as mitral E and A velocities, E deceleration time, E/A ratio, and mitral annular tissue Doppler septal Es’ and El' velocities. Each examination featured real-time calculation of EF and global longitudinal strain. Blood pressure measurements were taken upon the completion of the echocardiography assessment. Myocardial work index parameters, apical and basal rotation, twist, left atrial and right atrial strain, and EF, as well as right ventricular strain, were computed offline.

Statistical methods

Descriptive statistics were computed to summarize each parameter's characteristics at each time point. Continuous data were presented as means ± standard deviations. The normal distribution of all differences was assessed using the Kolmogorov-Smirnov test. A two-tailed, dependent T-test was utilized for continuous variables. Categorical data were reported as numbers and percentages. Univariate analysis was conducted through the chi-square test or Fisher’s exact test, as appropriate, to determine the significant variables (p<0.05). Correlation analysis was performed using Pearson's correlation coefficient. The statistical analysis was conducted using Statistical Product and Service Solutions (SPSS) (version 28.0; IBM SPSS Statistics for Windows, Armonk, NY).

Ethical approval 

The study was approved by the Ethics (Helsinki) Committee at Shamir (Assaf Harofeh) Medical Center for the conduct of clinical studies (0217-20-ASF) and at Kaplan Medical Center (KMC-21-0076). All methods were conducted following the relevant guidelines and regulations.

## Results

The study comprised a total of 25 participants, with a mean age of 52.4±9.3 years. All enrolled individuals were women and possessed no prior history of cardiac disease, as indicated in Table 1.

**Table 1:** General patients’ baseline characteristics ACEI: angiotensin-converting enzyme inhibitors; ARBs: angiotensin II receptor blockers; CAD: coronary artery disease

Variable	Value
Number of patients	25
Age, years	52.4±9.3
Weight, kg	71.8±13.4
Hypertension	6 (24%)
Family history of CAD	3 (12%)
Hypercholesterolemia	10 (40%)
Diabetes mellitus	1 (4%)
Smoker	3 (12%)
Statins	2 (8%)
ACEI/ARBs	5 (20%)
Beta-blockers	6 (24%)

Among these patients, six individuals (24%) had a medical history of hypertension and were managed pharmacologically with beta-blockers and angiotensin-converting enzyme inhibitors (ACEI)/angiotensin II receptor blockers (ARBs). Each of the patients underwent a comprehensive regimen that encompassed breast surgery, irradiation, and chemotherapy, as outlined in Table 2.

**Table 2:** Surgery, chemotherapy, and irradiation therapy TCH-P: docetaxel carboplatin, trastuzumab, and pertuzumab

Variable	N	%
Adriamycin	14	56
TCH-P	10	40
Cyclophosphamide	8	32
Trastuzumab	10	40
Pertuzumab	8	32
Carboplatin	3	12
Paclitaxel	18	72
Capecitabine	2	8
Irradiation	25	100
Surgery	25	100
Cardiac side effects		
Palpitations	2	8
Chest pain	2	8
Heart failure	3	12
Atrial fibrillation	1	4

Among the participants, eight individuals (32%) encountered cardiac symptoms, including palpitations in two cases, chest pain in two cases, heart failure in three cases, and atrial fibrillation in one instance. Notably, sinus rhythm was restored via cardioversion for the patient with atrial fibrillation. Table 3 encapsulates the pertinent echocardiography data obtained throughout the study.

**Table 3:** Echocardiography data before, during, and after chemotherapy BSA: body surface area; SBP: systolic blood pressure; DBP: diastolic blood pressure; HR: heart rate; EFa: Automatic EF; EFs: EF measured with Simpson method; EDVi: end-diastolic volume index; ESVi: end-systolic volume index; LVMi: left ventricular mass index; LAVi: left atrial volume index; GLS: global longitudinal strain; GWI: global work index; GCW: global constructive work; GWW: global wasted work; GWE: global work efficacy; E dec: deceleration slop of E wave; AR: apical rotation; BR: basal rotation; TAPSE: tricuspid annulus systolic velocity; TAPSV: tricuspid annulus Tissue Doppler velocity; RVGS: right ventricular global strain; RVFWS: right ventricular free wall strain; LA Strain: Left atrial reservoir strain; LAEF: left atrial ejection fraction; RA strain: right atrial reservoir strain; RAEF: right atrial ejection fraction. p1: p-value of results at first echo exam versus second echo exam; p2: p-value of results at second echo exam versus third echo exam; p3: p-value of results at third echo exam versus first echo exam

Timing	Before chemotherapy	During chemotherapy	Post chemotherapy	p1	p2	p3
BSA, m2	1.76±0.16	1.76±0.17	1.76±0.17	0.88	0.94	0.82
SBP, mmHg	125.0±22.9	119.7±22.3	123.6±22.4	0.3	0.54	0.66
DBP, mmHg	72.4±11.6	70.9±16.8	72.7±13.9	0.79	0.68	0.85
HR, bpm	70.5±10.7	75.5±12.3	70.4±10.5	0.13	0.13	0.99
EFa, %	59.0±3.9	56.8±5.0	57.1±5.0	0.08	0.82	0.12
EFs, %	62.1±5.3	59.9±5.2	59.4±5.1	0.15	0.73	0.075
EDVi, ml/m2	46.8±10.7	49.3±13.3	49.8±12.3	0.48	0.95	0.4183
ESVi ml/m2	17.9±4.1	19.5±5.3	20.9±5.5	0.19	0.39	0.037
LVMi, g/m2	75.0±18.2	78.6±17.7	78.4±20.3	0.57	0.97	0.62
LAVi, ml/m2	28.2±6.5	27.8±6.2	28.9±6.9	0.73	0.55	0.8
GLS, %	-19.6±1.6	-18.5±2.3	-18.5±2.2	0.05	0.46	<0.05
GWI, mmHg%	2,016.0±477.2	1,845.1±333.0	1,925.1±371.8	0.14	0.43	0.43
GCW, mmHg%	2,322.9±514.4	2,123.4±387.2	2,191.6±389.0	0.11	0.54	0.27
GWW, mmHg%	66.56±30.0	77.3±46.6	72±41.6	0.31	0.67	0.56
GWE, %	96.4±1.2	95.9±1.7	95.9±1.9	0.15	0.94	0.21
E/E'	7.0±1.9	7.0±1.5	7.0±1.9	0.87	0.99	0.9
Edec, msec	161.9±44.8	159.0±49.5	185.5±54.9	0.47	0.08	0.22
E/A	1.1±0.4	1.0±0.3	1.0±0.3	0.47	0.95	0.2
TWIST, °	18.8±7.8	17.3±5.8	15.6±7.4	0.42	0.37	0.13
AR, °	13.1±8.3	12.7±5.1	10.1±5.0	0.83	0.068	0.12
BR, °	-4.1±5.3	-4.9±5.2	-5.5±5.1	0.58	0.69	0.34
TAPSE, cm	2.0±0.3	2.0±0.3	2.1±0.3	0.61	0.25	0.5
TAPSV, cm/s	13.0±1.9	12.4±2.0	12.1±2.3	0.25	0.65	0.13
RVGS, %	-20.3±3.2	-19.1±2.7	-19.4±3.5	0.14	0.66	0.36
RVFWS, %	23.5±4.7	-21.9±4.4	-22.8±4.7	0.22	0.46	0.6
LA Strain, %	31.1±7.8	28.4±5.9	28.0±5.7	0.18	0.09	0.11
LAEF, %	58.7±10.5	57.2±8.5	55.5±8.3	0.57	0.47	0.23
RA strain, %	33.8±8.1	30.0±8.4	31.0±7.4	0.11	0.66	0.21
RAEF, %	49.3±13.8	45.4±9.4	45.4±10.3	0.23	0.94	0.27

Across the echocardiography examinations conducted at baseline, during chemotherapy (17±6 weeks from the start), and post-chemotherapy (25±9 weeks after the completion), there were no notable differences in conventional and advanced echocardiography parameters, with one exception - GLS. Specifically, GLS exhibited a decrease during chemotherapy compared to the baseline, demonstrating borderline significance 18.5±2.3 versus -19.6±1.6 (p=0.05). This trend persisted after the completion of chemotherapy, where GLS remained diminished relative to the baseline examination, as evidenced by values of -18.5±2.2 versus -19.6±1.6 (p<0.05). We further categorized a subset of 15 patients in whom there was a demonstrable decrease in GLS before chemotherapy. The data for this subgroup have been systematically examined and are presented separately in Table 4.

**Table 4:** Left ventricular function parameters of patients in whom global longitudinal strain was reduced following chemotherapy GLS: global longitudinal strain; EFs: EF measured by Simpson equation; Efa: EF measured automatically; GWI: global work index; GCW: global constructive work; GWW: global wasted work; GWE: global work efficacy. p1: p-value of results at the first echo exam versus second echo exam; p2: p-value of results at second echo exam versus third echo exam; p3: p-value of results at third echo exam versus first echo exam

Echo parameters	Timing of echocardiography examination relative to chemotherapy			p1	p2	p3
	Before	During	Post			
GLS, %	-19.9±1.4	-17.4±1.9	-18.6±2.1	0.0006	0.1	0.0498
EFs, %	62.9±5.6	58.1±4.6	58.7±4.2	0.01	0.68	0.028
EFa, %	60.07±3.4	54.73±4.9	56.6±5.11	0.0017	0.31	0.038
GWI, mmHg%	1,864.5±389.6	1,789.4±261.1	1,879.5±306.2	0.54	0.39	0.91
GCW, mmHg%	2,184.6±417.6	2,052.2±300.0	2,143.1±348.6	0.33	0.64	0.77
GWW, mmHg%	52.7±25.2	78.4±42.6	65.0±46.3	0.05	0.42	0.37
GWE, %	96.8±1.3	95.6±1.9	96.3±1.9	0.05	0.3	0.44

Notably, upon individual analysis, GLS exhibited a significant decline in relation to the baseline values, registering at 17.4±1.9 compared to 19.9±1.4 (p=0.0006). Furthermore, a significant decrease in EF was observed following chemotherapy, showing a reduction from 62.9±5.6% to 58.1±4.6% (p=0.01). Throughout the course of chemotherapy, there was a noteworthy increase in global wasted work, a trend that achieved borderline significance. In tandem, global work efficacy experienced a decline with borderline significance (p=0.05), as outlined in Table 4. We conducted a comparative analysis between two distinct groups within the study: one comprised 15 patients (60%) exhibiting a decrease in GLS during chemotherapy, and the other consisted of 10 patients (40%) in whom GLS remained unchanged. The results of this comparison are summarized in Table 5.

**Table 5:** Comparative clinical and echo data of patients in whom GLS was reduced following chemotherapy versus patients with normal GLS *Group 1: patients in whom the strain was reduced following chemotherapy; **Group 2: patients wherein the strain did not change during chemotherapy; ***Symptoms: chest pain, palpitations, heart failure; ACEI: angiotensin-converting enzyme inhibitors; ARBs: angiotensin receptor blockers; TCH-P: docetaxel carboplatin, trastuzumab, and pertuzumab; SBP: systolic blood pressure; DBP: diastolic blood pressure; LVMi: left ventricular mass index; EFs: EF measured with Simpson method; EFa: EF measured automatically; GLS: global longitudinal strain; GWI: global work index; GCW: global constructive work; GWW: global wasted work; GWE: global work efficacy

Variable	*Group 1	**Group 2	P value
Number of patients	15 (60%)	10 (40%)	NA
Age, years	52.2±11.0	52.6±4.9	0.9
***Symptoms	3 (20%)	4 (40%)	0.44
Hypertension	1 (6.7%)	5 (50%)	0.017
Baseline medications			
ACEI and ARBs	1 (6.7%)	4 (40%)	0.02
Beta-blockers	2 (13.3%)	4 (40%)	0.2
Chemotherapy medications			
Adriamycin	8 (53.3%)	6 (60%)	0.96
TCH-P	7 (46.7%)	3 (30%)	0.86
Cyclophosphamide	4 (26.7%)	4 (40%)	0.66
Trastuzumab	8 (53.3%)	2 (20%)	0.55
Pertuzumab	6 (40%)	2 (20%)	0.59
Carboplatin	2 (13.3%)	1 (10%)	0.77
Paclitaxel	12 (80%)	6 (60%)	0.44
SBP at first echo, mmHg	117.1±17.9	140.7±24.0	0.02
DBP, mmHg	66.1±7.7	80.8±11.9	0.005
LVMi, g/m2	72.0±13.7	81.2±23.7	0.3
EFs base, %	62.9±5.6	60.8±4.7	0.33
EFa base, %	60.1±3.4	57.6±4.5	0.17
GLS, %	-19.9±1.4	-19.0±1.7	0.2
GWI, mmHg%	1,864.5±389.6	2,251.6±487.8	0.06
GCW, mmHg%	2,184.6±417.6	2,556.2±542.3	0.1
GWW, mmHg%	52.7±25.2	86.0±23.7	0.004
GWE, %	96.8±1.3	96±0.6	0.05

Interestingly, no notable disparities were identified in terms of age, body size, symptoms, type of chemotherapy administered, or baseline conventional echocardiography parameters between the two groups. However, patients in the subgroup where GLS remained consistent throughout chemotherapy displayed a higher prevalence of hypertension (50% versus 6.7%), which yields a statistically significant difference (p=0.017). Furthermore, this subgroup was more frequently subjected to treatment involving ACEI/ARBs (40% versus 6.7%), which reveals a statistically significant distinction (p=0.02). These patients exhibited elevated systolic blood pressure during the baseline echocardiography examination, registering at 140.7±24 compared to 117.1±17.9 in the group where GLS did not change (p=0.02). Additionally, diastolic blood pressure also demonstrated significant differences (as outlined in Table 5). As an ancillary outcome, global wasted work was markedly higher in patients with unchanged GLS during chemotherapy, with values of 86.0±23.7 compared to 52.7±25.2 in the contrasting group (p=0.004). Within the scope of this study, a notable correlation was established between the Simpson-based measurement of EF and the automated calculation of EF derived from speckle-tracking imaging (Table 6).

**Table 6:** Pearson Correlation between biplane Simpson and automatic speckle tracking imaging-based calculation of EF and volumes CI: confidence interval; EF: ejection fraction; EDV: end-diastolic volume; ESV: end-systolic volume

Variable	Correlation	P value	Count	CI
EF	0.50	<0.001	75	95%
EDV	0.73	<0.001	75	95%
ESV	0.59	<0.001	75	95%

**Figure 1:**
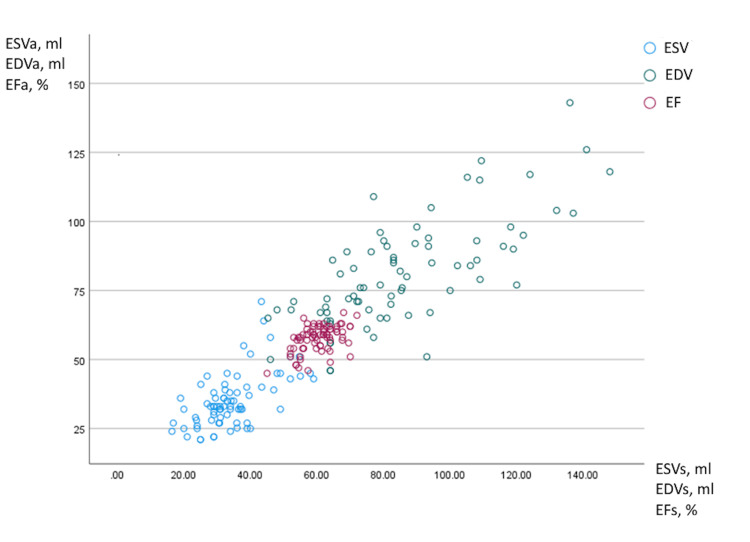
Correlation between two methods of EF calculation: automatic and Simpson method X-axis: Simpson method-based calculation of ESVs end-systolic volume; EDVs: end-diastolic volume; EFs ejection fraction; Y-axis: automatic speckle tracking imaging-based calculation of ESVa end-systolic volume, EDVa end-diastolic volume, and EFa ejection fraction; ESV: blue color; EDV: green color; EF: pink color

This correlation is visually depicted in Figure 1.

## Discussion

The results of the study show several interesting findings. We observed a significant decrease in GLS during and after chemotherapy when compared to baseline values. This decrease was particularly notable in a subgroup of patients (15 out of 25), where the reduction in GLS was more pronounced and significant. Our study also revealed a significant decrease in EF following chemotherapy, which is in concordance with previous studies. Additionally, the changes in global wasted work and global work efficacy, albeit with borderline significance, indicate alterations in cardiac mechanics during chemotherapy. Previous studies also have yielded conflicting outcomes regarding the supplementary influence of myocardial work index parameters, with findings ranging from modest [20], encouraging [21], and to their potential utility in specific scenarios [22]. A notable association was found between patients whose GLS did not change during chemotherapy and a history of hypertension. This suggests that preexisting hypertension might have a protective effect on myocardial function during chemotherapy. Patients with unchanged GLS were more often on medications such as ACEI/ARBs, which might have contributed to preserving myocardial function under the stress of chemotherapy. The absence of significant changes in twist and rotation indicates that the twisting motion of the left ventricle was maintained throughout the treatment and the overall twisting mechanics of the left ventricle were not compromised. Right ventricular (RV) function parameters, including tricuspid annular plane systolic excursion (TAPSE) and RV tissue Doppler velocities, and RV strain were unchanged. The stable left and right atrial strain and EF suggest that the left and right atrial function was not significantly affected by the treatment regimen. The lack of significant changes in diastolic parameters such as the E/E' ratio, E/A ratio, and E deceleration suggests that the diastolic function remained relatively stable throughout the treatment. Overall, the stability of these parameters in our study could be interpreted in several ways: the treatments might predominantly affect certain areas of the heart, such as the left ventricle, while sparing others, such as the right ventricle and atria. These parameters might change over a longer follow-up period, indicating the need for continued monitoring of these patients’ cardiac health. While these parameters did not show significant changes, it is crucial to recognize that their stability does not diminish the significance of the changes observed in GLS and EF. GLS and EF appear to be particularly sensitive markers for cardiac effects in this patient population. The lack of changes in other parameters could potentially be explored further in future studies to better understand the complex interactions between different cardiac structures and functions in the context of cancer treatments.

The study revealed a strong correlation between EF measurements obtained through the biplane Simpson method and those derived using an automatic EF calculation. This correlation indicates a promising alignment between the manual and automated methods for assessing EF in this patient group. The biplane Simpson method demonstrated concurrence with the automated measurement, which suggests that the latter could potentially serve as a reliable alternative for EF assessment in breast cancer patients.

A sample size of 25 patients might limit the statistical power of our study and its ability to detect subtle changes or associations. A larger sample could provide more robust results. Our study was conducted at two medical centers; hence, the findings may not fully represent the diversity of patient populations and treatment practices observed across different centers. The effect of specific chemotherapeutic agents was not an objective of our study.

## Conclusions

The impact of chemotherapy on breast cancer patients' cardiac function is notably manifested through reductions in EF and GLS, rather than discernible changes in myocardial work index parameters, twist, and diastolic function. Moreover, our study contributes to the understanding of EF measurement techniques in clinical practice. We found a strong correlation between EF measurements obtained through the biplane Simpson method and those computed using an automatic EF calculation. This suggests that both methods are reliable and can be interchangeably used for assessing EF in breast cancer patients undergoing chemotherapy.

Furthermore, our findings suggest potential avenues for cardioprotective strategies in breast cancer patients undergoing chemotherapy, particularly among those with hypertension and specific medications. The observation that these patients exhibited less pronounced changes in GLS indicates that certain antihypertensive medications, such as ACE inhibitors and ARBs, may confer cardioprotective benefits during chemotherapy. Further research is warranted to validate these findings and explore additional cardioprotective strategies, ultimately improving the cardiac care and outcomes of breast cancer patients undergoing chemotherapy.
